# Distribution of Aquatic Vertebrates in the Winter Dry Season Informing Water Resource Management in a Large Floodplain Lake

**DOI:** 10.3390/biology15080611

**Published:** 2026-04-13

**Authors:** Hui Wang, Zijun Wu, Yanping Zhang, Jinfeng He, Guodong Ding, Chenhong Li, Haixin Zhang

**Affiliations:** 1 Jiangxi Provincial Fishery Research Institute, Nanchang 330039, China; 2 Engineering Research Center of Environmental DNA and Ecological Water Health Assessment, Shanghai Ocean University, Shanghai 201306, China; 3 Jiangxi Fishery Resources and Ecological Environment Monitoring Center, Nanchang 330039, China; 4 Shanghai Universities Key Laboratory of Marine Animal Taxonomy and Evolution, Shanghai Ocean University, Shanghai 201306, China

**Keywords:** floodplain lake, environmental DNA, hydroacoustics, community assembly, water resource management

## Abstract

Hydrological fluctuations drive community dynamics in floodplain lakes, yet their integration into water resource management remains limited. Here, we integrated environmental DNA (eDNA) metabarcoding with hydroacoustic surveys to investigate vertebrate community assembly in China’s largest freshwater lake (Poyang Lake) during the winter dry season. We detected 65 vertebrate species, with Cypriniformes dominating. Beta-diversity partitioning revealed that turnover dominated taxonomic and functional dissimilarity, while phylogenetic beta diversity was characterized by nestedness, which is consistent with environmental filtering. Functional richness declined with water depth, coinciding with hydroacoustic vertical size stratification, indicating niche partitioning along depth gradients. Null model analysis showed stochastic processes (ecological drift) dominated regional assembly (72.97%), whereas joint species distribution modeling attributed explained variation to environmental factors (28.9%, notably water depth) and species associations (29.7%) at local scales. This hierarchical framework, regional stochasticity shaping the species pool and local deterministic filtering structuring communities, reframes environmental flow conceptualization: effective management must preserve the full spectrum of hydrological variability and maintain bathymetrically diverse habitats that support functional niche differentiation. The integrated eDNA-hydroacoustic approach offers a non-invasive, high-resolution toolkit for biological assessment within regulatory water quality frameworks.

## Simple Summary

Floodplain lakes are biodiversity hotspots, yet understanding how water level changes shape species communities remains limited. Using eDNA technology and sonar, we studied species in China’s largest freshwater lake without catching them. Results revealed that random chance largely determines which species live in the lake overall, but water depth acts as a local filter that sorts species by body size and ecological roles. This means protecting lake biodiversity requires two things: maintaining natural water level fluctuations and preserving areas with varying depths. Managers can use these insights to set better environmental flow targets and design more effective conservation strategies for floodplain lakes worldwide.

## 1. Introduction

Understanding the relative ecological processes that drive community assembly and maintain biodiversity is the core mission of community ecology [1,2]. Rivers and lakes are important components of the water cycle and a significant source of freshwater, while also maintaining the stability of freshwater ecosystems [3,4]. Connected lakes, as key elements of the river–lake continuum, typically harbor high biodiversity and provide vital ecosystem services. Their community dynamics are regulated by the interplay of hydrological rhythms, particularly seasonal water level fluctuations, and habitat heterogeneity, such as variations in water depth and topography [5]. Fish, which account for a quarter of global vertebrates, are a crucial component of aquatic ecosystems and an ideal subject for studying community assembly [6].

Community assembly has been the central topic of community ecology and conservation biology, which refers to species colonization processes from regional metacommunities to local ecosystems, elucidating mechanistic determinants of biodiversity patterns [7,8]. The mechanisms of community construction have been summarized in various aspects by various theories. Niche-based theory usually describes local, niche-based processes such as environmental filtering and species interactions [9]. Meanwhile, neutral theory more emphasizes the importance of spatial dynamics and ecological drift [9]. Modern ecology holds that the relative importance of niche and neutrality depends on environmental context, demanding considerations of how the importance changs across scales [10,11]. This hierarchical perspective is unified by metacommunity theory, which integrates different scales by viewing community aggregation as a function of the interaction between local conditions (e.g., environmental factors, species associations) and regional dynamics (e.g., dispersal) [10,12]. According to the prevailing synthesis, these complex dynamics can be deconstructed into four fundamental processes: selection, drift, dispersal, and speciation [1,2]. Niche-based deterministic filters interact synergistically with neutral stochastic processes to collectively regulate biodiversity configuration in different ecological scales [10,13,14].

Disentangling the relative contributions of regional species pools and local assembly processes constitutes a foundational challenge in community ecology [15,16]. The regional pool, shaped by biogeographic history and evolutionary dynamics, establishes the broad template of potential colonists, while local environmental heterogeneity then acts primarily by filtering this regional template, modulating which subsets of species can successfully establish and persist within specific habitats [17,18]. The processes governing biodiversity, including dispersal limitation, environmental filtering, and biotic interactions, interact across spatial and temporal scales, generating complex, emergent patterns. For example, high dispersal rates can homogenize communities and override historical priority effects, thereby amplifying the detectable signal of biotic interactions at larger scales, whereas strong dispersal limitation may locally mask such interactions [19]. Community beta diversity (β-diversity) serves as an integrative metric of these cross-scale dynamics [15]. It quantifies compositional variation among sites, thereby capturing the net outcome of the hierarchical interplay between regional species availability, local abiotic and biotic filters, and stochastic demographic forces [20,21].

Floodplain lakes provide critical ecosystem services including flood regulation, water purification, fishery support, and biodiversity maintenance, yet their ecological integrity is increasingly threatened by anthropogenic hydrological alterations, dam construction, water abstraction, and climate change, that disrupt natural flow regimes [5,22,23,24]. Understanding how hydrological fluctuations shape aquatic communities is therefore essential for setting environmental flow targets, designing lake restoration strategies, and implementing bioassessment programs under regulatory frameworks such as the EU Water Framework Directive, the U.S. Clean Water Act, and China’s Water Ten Plan [25,26,27]. However, the translation of hydrological data into ecologically meaningful management thresholds remains a persistent challenge, largely due to insufficient mechanistic understanding of how communities respond to hydrological variation across spatial scales [28].

Poyang Lake, the largest freshwater lake in China, is a critical ecotone within the Yangtze River floodplain system and characterized by dynamic river–lake interactions that cause significant seasonal fluctuations in water levels, creating an environment ranging from high randomness to strong environmental gradients [29]. Traditional approaches for assessing species communities have provided valuable insights but can be limited in their ability to fully capture community complexity [30]. In contrast, the integration of hydroacoustic detection and environmental DNA (eDNA) metabarcoding leverages their complementary strengths. Hydroacoustic technology provides high-resolution data on species density and habitat usage. Combining this with eDNA taxonomic assignment could result in highly sensitive species diversity and density detection [31,32,33]. Hence, this integrated approach allows us to: (1) reveal the composition of species community in Poyang Lake during the winter dry season; (2) assess the relative importance of deterministic versus stochastic processes at both overall and local scales; and (3) quantify the specific contributions of abiotic factors (such hydrological drivers) and biotic interactions to species community, deriving actionable insights for water resource management.

## 2. Materials and Methods

### 2.1. Study Area, Collection of Water Samples and Hydroacoustic Data

**Figure 1:**
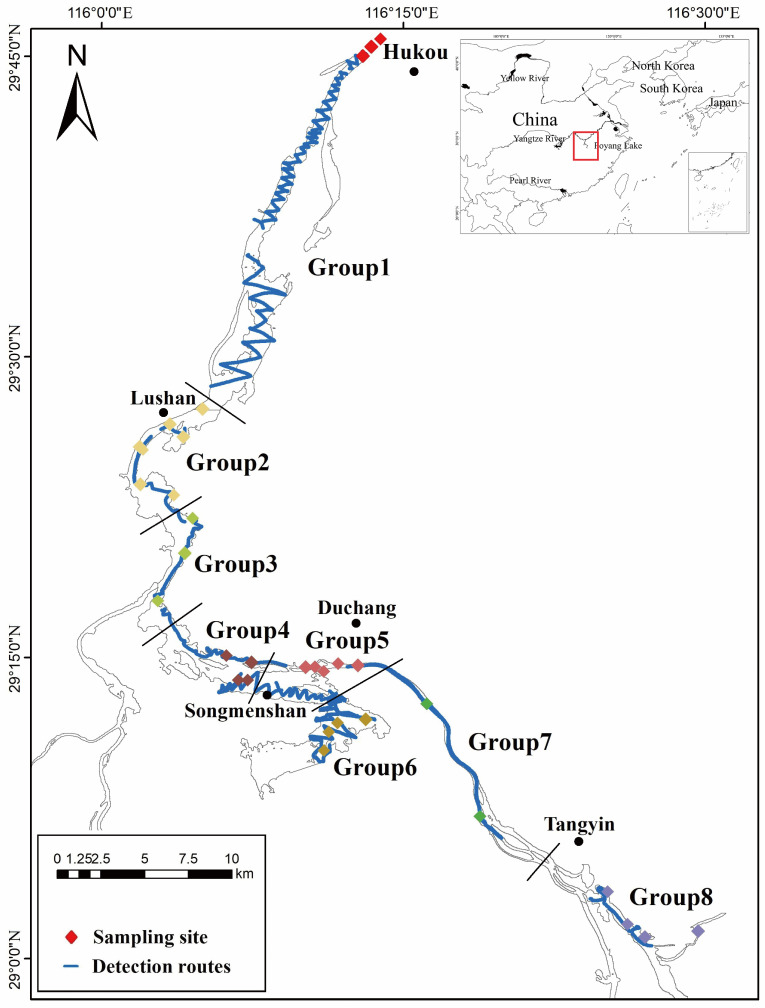
Sampling sites in December in Poyang Lake. Different colors represent different groups.

Poyang Lake (28°22′~29°45′ N, 115°47′~116°45′ E), China’s largest seasonal freshwater lake and a crucial Asian wetland ecosystem, exhibits dramatic hydrological fluctuations (expanding to >4000 km^2^ during flood seasons while contracting to <1000 km^2^ in dry periods). We collected eDNA samples from 34 systematically distributed stations across the lake during the winter dry season (6–13 December 2023). Sampling stations were clustered into 8 hydrological groups based on spatial proximity and connectivity (Figure 1). At each station, a 2 L integrated water sample was obtained through vertical column sampling (underwater 1.5 m) using an upright water collector, with thorough homogenization of water column strata. All sampling equipment was UV-sterilized and pre-treated with 10% bleach solution. We filtered water samples in situ through 0.45 µm mixed cellulose ester membranes (Merck Millipore, Burlington, MA, USA). Membranes were immediately preserved in CTAB buffer (Solarbio, Beijing, China) at liquid nitrogen during fieldwork and transferred to −80 °C storage. Hydroacoustic assessment utilized a Biosonics DT-X split-beam scientific echosounder (200 kHz; BioSonics Inc., Seattle, WA, USA) with a standardized “Z”-transect sampling design (Figure 1). The transducer (6.8° nominal beam angle) was deployed at 0.5 m depth via a starboard-mounted stabilization frame, maintaining vessel speed ≤ 10 km/h throughout surveys to control noise. System calibration followed protocols using 38.1 mm tungsten carbide reference spheres from [34]. Acoustic parameters included: 5 pings/s repetition rate, 0.4 ms pulse duration, and −130 dB TS threshold. Raw data acquisition utilized the BioSonics Visual Acquisition 5.0 software with automatic noise filtration (threshold: −70 dB). Chlorophyll-a concentrations were derived from Landsat8 OLI/TIRS imagery accessed via USGS EarthExplorer (https://earthexplorer.usgs.gov/, accessed on 30 December 2024) by using a machine learning algorithm [35].

### 2.2. Molecular Experiment

Given the complex composition of eDNA and potential inhibitors (e.g., humic acids), DNA extraction was performed with a modified phenol–chloroform protocol under UV-sterilized conditions. Membranes were digested with 20 μL proteinase K (20 mg/mL; Yeasen Biotech, Shanghai, China) at 56 °C for 1 h, followed by organic phase separation via centrifugation (16,000× *g*, 10 min) with 2 mL chloroform. The aqueous supernatant was precipitated using 500 μL ice-cold isopropanol and 250 μL 5 M NaCl, pelleted by centrifugation (14,000× *g*, 15 min) and washed twice with 70% ethanol. Purified DNA was resuspended in 50 μL TE buffer (10 mM Tris-HCl, 1 mM EDTA, pH 8.0) and quantified via the Qubit 4.0 Fluorometer (Thermo Fisher Scientific, Waltham, MA, USA). A 171 bp fragment of the mitochondrial 12S rRNA gene was amplified using tele02 primers (F: 5′-AAACTCGTGCCAGCCACC-3′; R: 5′-GGGTATCTAATCCCAGTTTG-3′) [36] following the thermocycling protocol from [37]. PCR products were purified using AMPure XP magnetic beads (Beckman Coulter, Brea, CA, USA) and eluted in 20 μL TE buffer. Library preparation included dual-indexed Illumina adaptor ligation during a second PCR (8 cycles), followed by size selection via 2% agarose gel electrophoresis. Target bands were extracted using the FastPure Gel DNA Extraction Mini Kit (Vazyme Biotech, Nanjing, China) and sequenced on an Illumina NovaSeq 6000 platform (San Diego, CA, USA, 2 × 150 bp paired-end reads).

### 2.3. Hydroacoustic Data Analysis

Echo signal processing was conducted in Visual Analyzer 4.1 (BioSonics Inc., Seattle, WA, USA) with the following thresholds: single echo detection threshold of −70 dB; echo length range from 0.75 to 3.0; time-varied gain of 40 lgR; minimum target spacing of 2 pings; and minimum target count per track of 3 pings. Survey coverage (D) was calculated as $D=\frac{L}{\surd A}$, where *L* represents transect length (m), and *A* represents survey area (m^2^) [38]. Target Strength (TS)-to-Length conversion used TS = 20logTL − 71.9, where *TS* means target strength (dB) and *TL* means total species length (cm). To estimate species density (per 3000 pings/sampling unit; *n* = 38), the density of each sampling unit is used to calculate the average density for the entire survey area. This is combined with the volume of the water body in the survey area to estimate species density and resources by $\rho_{i}=\frac{S_{i}\times 1000}{V_{i}}$, where $\rho_{i}$ represents species density in *i*th unit (ind./1000 m^3^), $S_{i}$ represents species count per unit and $V_{i}$ means volume in *i*th unit. Vertical stratification divided the water column into three layers, upper (0~33% depth), middle (33~66% depth) and lower (66~100% depth). Statistical analyses (ANOVA, spatial autocorrelation) were performed in SPSS 25.0 (IBM Corp., Armonk, NY, USA) and ArcGIS 10.2 (ESRI), with density distributions visualized via inverse distance weighting (IDW) interpolation (power = 2, search radius = 500 m).

### 2.4. Sequencing Data Processing and Statistical Analysis

Raw paired-end sequencing data underwent quality control through Fastp v0.23.2 [39] with adapter trimming and removal of low-quality reads (Phred score < 20, length < 50 bp). Overlapping reads were merged using PANDAseq v2.11 [40] with a minimum overlap of 20 bp and 95% sequence identity. Demultiplexing was performed via sample-specific dual-index barcodes, followed by length filtering (retaining sequences within ±10% of the expected 171 bp target). Chimeric sequences and PCR repeat amplification templates were removed using VSEARCH v2.18.0, generating amplicon sequence variants (ASVs) with the UNOISE3 algorithm [41]. Taxonomic assignment of ASVs was conducted via BLASTn v2.13.0 against a curated reference database with 3236 genera and 11,236 species combining the NCBI nt, MitoFish database and self-sequenced mitochondrial genomes [42,43,44]. Matches were filtered at 97% identity with over 90% query coverage, with hierarchical classification resolving ambiguous assignments.

To characterize the relationship between species richness and hydroacoustic densities (ind./1000 m^3^), we employed generalized additive models (GAMs; mgcv v1.9-0) with thin-plate regression splines (*k* = 3) to accommodate potential non-linearity. Additionally, Spearman correlation analysis was applied to hydroacoustic data (uniformly grouped) to examine the associations among the coefficient of variation (CV) of density, average density, and the CV of water depth. Taxonomic, phylogenetic, and functional β-diversity were computed using the Sørensen index via the betapart package (v1.6) [45]. Phylogenetic relationships among the detected species were inferred from whole mitochondrial genomes. Key functional traits, including trophic group, spawning substrate, vertical habitat, and maximum body length, were compiled from databases and the literature to construct a functional trait matrix. Functional richness (FRic) and functional divergence (FDiv) were subsequently calculated using the fundiversity package (version 1.1.1) [46], and their relationships with water depth and river width were assessed using GAMs. For a better understanding of the assembly process, stochastic/deterministic processes were assessed via a neutral model developed by Sloan et al. (2006) through MicEco (https://github.com/Russel88/MicEco, accessed on 20 November 2024) and βNTI (β-nearest taxon index) through iCAMP v1.5.1 [47,48]. βNTI>1.96 indicates heterogeneous selection while βNTI<−1.96 indicates homogeneous selection, both representing deterministic processes. Since |βNTI|≤1.96 indicates that the community is primarily influenced by stochastic processes, it is further partitioned by the Raup–Crick index (RC) [49]. RC>0.95 indicates dispersal limitation, RC<−0.95 indicates homogeneous dispersal, and |RC|≤0.95 indicates that the community is influenced by undominated processes such as ecological drift. Joint Species Distribution Models (jSDMs) were the statistical methods used in ecology to simultaneously analyze the distribution of multiple species in relation to environmental factors. Therefore, the occurrence matrix (presence/absence) and abiotic factors (chlorophyll-a (μg/L), water depth (m), river width (m) and spatial coordinates) were incorporated for understanding community-scale distribution patterns through a sjSDM [50]. To comprehensively assess the relative importance of abiotic factors (river width, river depth and chlorophyll concentration), we employed the hierarchical partitioning method based on GAM (permu.gamhp) due to this method not being affected by the collinearity and non-linear relationships of the variables. All figures generated using ggplot2 v3.4.2 [51] adhered to the ColorBrewer 2.0 perceptually uniform palettes.

## 3. Results

### 3.1. Community Composition and Size-Structured Vertical Stratification

**Figure 2:**
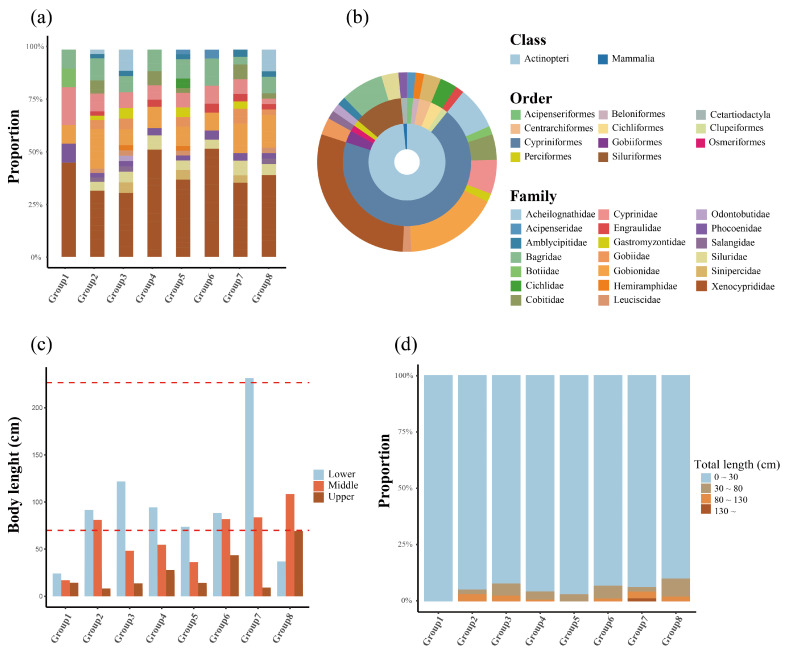
Community composition and vertical size stratification. (**a**) Taxonomic composition of fish assemblages per sampling group derived from eDNA, classified by family. (**b**) Overall taxonomic composition of the lake’s species community derived from eDNA, shown as proportional circles from class (innermost) to family (outermost). (**c**) Body length distribution of aquatic vertebrates across the water column (lower, middle, upper) derived from hydroacoustic data. The red dashed line indicates the body length range of *Neophocaena asiaeorientalis* (≥70 cm, according to GBIF https://www.gbif.org/species/4352040 accessed on 5 March 2026). (**d**) Proportional distribution of body length classes for each sampling group derived from hydroacoustic data.

Environmental DNA metabarcoding identified a vertebrate community of 65 species in Poyang Lake, which was overwhelmingly dominated by fishes from the order Cypriniformes (69.2%, *n* = 45) (Figure 2a,b). The families Xenocyprididae (29.2%, *n* = 19) and Gobionidae (16.9%, *n* = 11) were the most speciose, underscoring the lake’s status as a critical hotspot for cyprinid diversity. The critically endangered Yangtze finless porpoise (*Neophocaena asiaeorientalis*) was detected in all sampling groups, while two non-native cichlids (*Oreochromis niloticus* and *O. aureus*) exhibited a restricted, localized distribution (exclusive to Group 5). Four threatened species were documented in total, including the finless porpoise, the Chinese sturgeon (*Acipenser sinensis*), and the endemic provincial protected species *Rhynchocypris oxycephalus* and *Liobagrus anguillicauda*.

Hydroacoustic data revealed significant vertical stratification structured by body size (Figure 2c). Larger-bodied individuals predominantly occupied the lower and middle water layers across most groups. Group 8 was a notable exception, with its largest specimens found in the middle and upper layers. The community in Group 1 was characterized as miniaturized, comprising 11 species (through eDNA) with body lengths between 0~30 cm. Analysis of size–density relationships showed divergent patterns among groups; for instance, Group 2 exhibited an inverse relationship in its lower layer, where the longest species corresponded to the lowest densities (Figure 2d). In contrast, the lower layer of Group 7 supported the highest densities of the largest-bodied species, contributing to its dominant total density.

### 3.2. Taxonomic, Phylogenetic and Functional β-Diversity Partitioning

**Figure 3:**
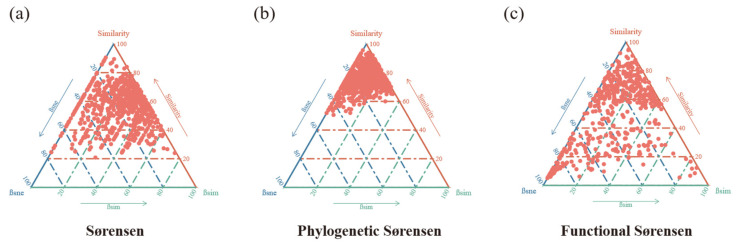
Partitioning of community dissimilarity. Beta diversity was decomposed into turnover (βsim, species/functional replacement) and nestedness (βsne, species/functional loss) components based on the Sørensen index for (**a**) taxonomy, (**b**) phylogeny, and (**c**) function.

We partitioned taxonomic, phylogenetic, and functional beta diversity to elucidate the mechanisms of community assembly (Figure 3). Species turnover (β_sim_) was the dominant component of both taxonomic (β_sim_: 0.817; β_sne_: 0.007) and functional (β_sim_: 0.763; β_sne_: 0.265) beta diversity, indicating that community differences primarily result from species and functional trait replacement across sites. In contrast, the introduction of phylogenetic relationships fundamentally shifted this pattern: phylogenetic beta diversity (β_Phy_) was dominated by the nestedness-resultant component (β_sne_), with turnover (β_sim_) decreasing substantially from 0.817 (β_Sør_) to 0.667 (β_Phy_). This significant decline in phylogenetic turnover provides strong evidence for phylogenetic niche conservatism, whereby coexisting species are more closely related than expected by chance, consistent with the action of environmental filtering on conserved traits.

### 3.3. Functional Diversity Responses to Environmental Gradients

**Figure 4:**
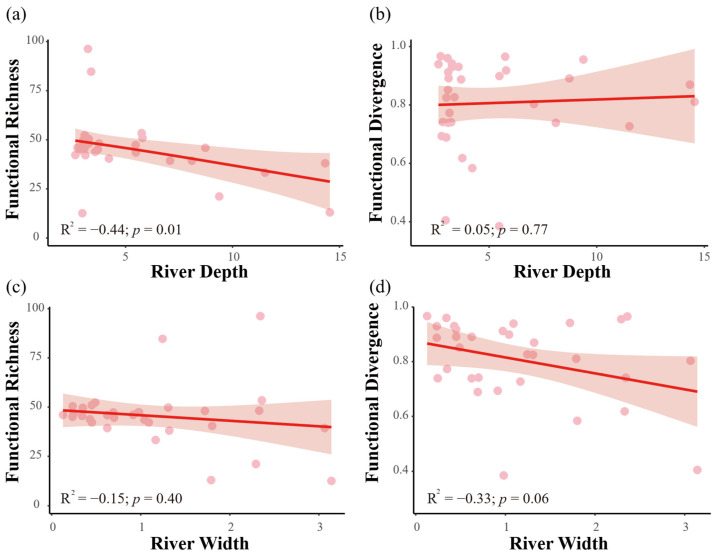
Functional diversity responses to environmental gradients. Relationships between (**a**) functional richness (FRic) and water depth, (**b**) functional divergence (FDiv) and water depth, (**c**) FRic and river width, and (**d**) FDiv and river width. The shaded areas represent 95% confidence intervals from the GAM fits. The significant decline in FRic with increasing depth (**a**) identifies water depth as a key hydrological variable modulating functional diversity, with implications for water level management.

The relationship between functional diversity and environmental gradients revealed a key signature of environmental filtering (Figure 4). Functional richness, representing the amount of trait space occupied by assemblages, exhibited a significant negative correlation with water depth (R = −0.44, *p* = 0.011), indicating that deeper waters support a narrower range of functional strategies (Figure 4a), suggesting that water depth acts as a key environmental filter shaping the functional composition of vertebrate communities. This relationship implies that water level drawdown, by reducing the availability of deeper habitats, could further compress the already limited functional niche space in shallow areas, potentially undermining ecosystem functions such as productivity and stability. Furthermore, the lack of a significant relationship between FRic and river width (R = 0.055, *p* = 0.77; Figure 4c) suggests that hydrological variables (specifically depth) are stronger drivers of functional diversity than geomorphological features in this system, underscoring the importance of water level management over channel morphology modification.

### 3.4. Community Stability, Assembly Processes, and Drivers of Species Distributions

**Figure 5:**
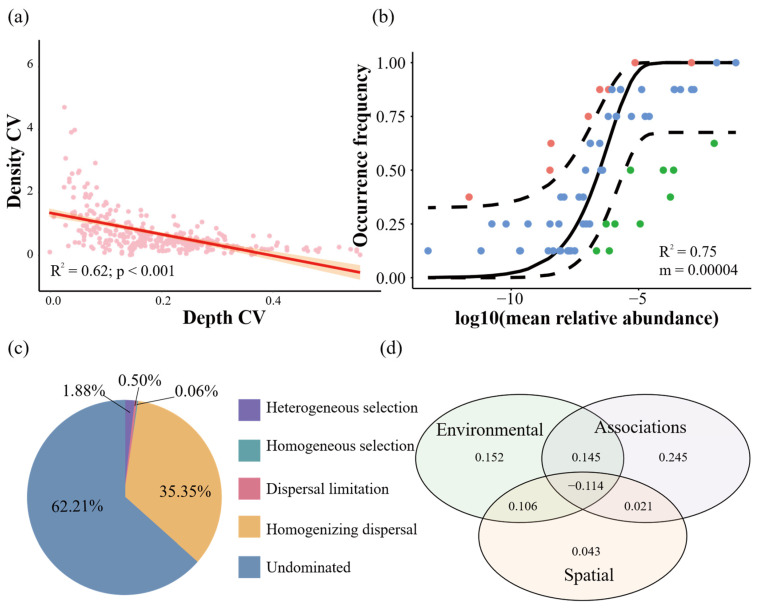
Community stability, assembly processes, and drivers of species distributions. (**a**) Relationship between the coefficient of variation (CV) of species density and average density. The red line represent regression curve. (**b**) Neutral model fitting according to [48]. The black dotted line indicates the 95% confidence interval. (**c**) Relative contributions of different ecological processes to local community assembly, as inferred from a null model (βNTI). “Undominated” primarily represents ecological drift. (**d**) Variance partitioning of species occurrences explained by environmental variables, species associations, and spatial structure. Despite regional-scale stochastic dominance (**c**), water depth emerges as a dominant environmental predictor in the jSDM (**d**), highlighting a locally manageable variable through which water resource managers can influence community structure.

Habitat heterogeneity, measured as the coefficient of variation (CV) in water depth, was a strong predictor of community stability. We found a significant negative correlation between the CV of water depth and the CV of species density (R^2^ = 0.62, *p* < 0.01; Figure 5a), indicating that more heterogeneous habitats support greater stability in species density distributions. This relationship was complemented by a weak, marginally non-significant positive trend between average density and depth CV (R^2^ = 0.1, *p* = 0.06). Neutral model analysis results (R^2^ = 0.75) indicated that the stochastic process has a greater impact on the community (Figure 5b). And null model analysis also revealed the overwhelming dominance of stochastic processes, with ecological drift and other undominated processes accounting for 72.97% of pairwise comparisons (Figure 5c). While this regional-scale stochasticity may appear to limit the predictability of community responses, the jSDM results (Figure 5d) demonstrate that the explained variation in species occurrences is nearly equally partitioned between environmental factors (28.9%) and species associations (29.7%). The results of hierarchical partitioning also show that water depth is the most prominent (Individual = 0.45, *p* = 0.005). Thus, water depth emerged as a dominant environmental variable within the jSDM, indicating that despite the overarching influence of stochastic processes at the regional scale, depth remains a locally manageable hydrological variable that exerts deterministic control on community structure. This finding suggests that water level regulation, by modulating depth gradients, can directly influence the realized composition of local assemblages, even if the regional species pool is shaped by broader-scale stochastic dynamics.

## 4. Discussion

The integration of eDNA metabarcoding with hydroacoustic profiling employed in this study represents an advanced, non-invasive toolkit for comprehensive aquatic biodiversity assessment [52]. Applying this integrated framework to Poyang Lake during the winter dry season, we investigated aquatic vertebrate species composition and elucidated the mechanisms underlying community assembly in Poyang Lake. A total of 65 species were detected, including four protected species, such as *Acipenser sinensis*, *Neophocaena asiaeorientalis*, *Rhynchocypris oxycephalus*, and *Liobagrus anguillicauda*, and two non-indigenous species, *Oreochromis niloticus* and *Oreochromis aureus* [53,54,55]. Therein, Cypriniformes constituted the dominant order, primarily represented by the families Cyprinidae and Cobitidae. Species such as *C. carpio* and *C. auratus* remain the major economic fishes in the lake [56,57]. Analyses of community dissimilarity revealed that species turnover was the dominant component of taxonomic β-diversity, indicating that spatial variation in assemblages is primarily driven by species replacement across heterogeneous habitats [58]. This pattern was further corroborated by incorporating phylogenetic information, which substantially reduced the overall β-diversity. The resulting phylogenetic clustering provides robust evidence for environmental filtering, whereby abiotic conditions selectively retain lineages with conserved, functionally analogous traits from the regional species pool [59,60,61]. Our data also demonstrate a significant decline in functional richness (FRic) with increasing water depth, concurrent with a rise in overall species density. Local biodiversity, mediated by differences in functional traits among organisms, can influence ecosystem properties such as stability [62]. The observed decline in FRic aligns with the vertical stratification of species body size documented via hydroacoustic data, reflecting niche partitioning that facilitates coexistence within a constrained functional niche space [63]. Such niche differentiation is central to species coexistence, as it allows organisms to allocate resources in ways that reduce competitive overlap, thereby supporting biodiversity maintenance.

Quantification of assembly drivers via a Joint Species Distribution Model (jSDM) attributed substantial and nearly equivalent proportions of variance to local environmental variables (28.9%) and species interactions (29.7%), underscoring the deterministic role of both abiotic filtering and biotic processes at the habitat scale. Paradoxically, a null model analysis (βNTI) revealed the overwhelming predominance of stochastic processes, predominantly ecological drift, at the whole-lake (regional) scale. This discrepancy aligns with theoretical perspectives on scale-dependency [64]. While biotic interactions are key determinants locally, their signature may diminish at broader scales [65]. Theoretical and empirical work suggests that in heterogeneous regional landscapes, environmental variability can mediate and dilute the net effect of species interactions, making stochastic processes like drift more prominent in shaping the regional species pool [65,66]. Thus, the strong deterministic signals at the local scale and the stochastic dominance at the regional scale are not contradictory; rather, they reflect the scale-dependent predominance of distinct assembly processes.

To mechanistically reconcile the apparent contradiction between local determinism and regional stochasticity, we invoke a cross-scale, hierarchical assembly framework [11,18]. At the regional scale, environmental stochasticity, exemplified by large-extent disturbances such as floods, acts as a potent random filter [67,68]. Historical and contemporary hydrological fluctuations stochastically filter and assemble the regional species pool, which subsequently serves as the source for local community assembly during periods such as the winter dry season, while factors like microclimate and microhabitat exert greater influence at finer spatial scales [68]. Here, hydrological contraction concentrates organisms, and water depth serves as a primary deterministic filter, selecting species based on their functional traits [69]. This scale-dependent interplay, where regional stochasticity sets the template for local deterministic filtering, aligns with reports of ecological drift predominating in other regional assemblages within Poyang Lake [70].

Our findings hold direct implications for the management of floodplain lakes subject to intensifying hydrological alteration. The hierarchical assembly framework fundamentally redefines how environmental flows should be conceptualized: preserving not merely average water levels but the full spectrum of hydrological variability, including seasonal fluctuations and interannual extremes, is essential to sustaining both regional species pools and local assembly processes [71,72]. Environmental flow targets should therefore incorporate metrics of hydrological dynamism, moving beyond fixed minimum flows alone. Furthermore, the significant decline in FRic with increasing depth, combined with hydroacoustically documented vertical size stratification, underscores depth gradients as critical determinants of functional niche space. Conservation planning must therefore prioritize bathymetrically diverse areas encompassing multiple depth strata, given that habitat homogenization through dredging or uniform deepening would collapse the niche differentiation mechanisms that underpin species coexistence [73,74]. The integrated eDNA-hydroacoustic approach presented here offers a non-invasive, high-resolution toolkit for operationalizing biological assessment within regulatory water quality frameworks, thereby enabling managers to detect ecological responses to hydrological alteration with unprecedented sensitivity. Nevertheless, several limitations warrant consideration. Our single-season snapshot represents only a temporal window of community dynamics, precluding assessment of seasonal or interannual variability in assembly processes. Moreover, while the integration of eDNA and hydroacoustics offers effective complementarity, the inherent limitations of each technique, such as the potential for eDNA diffusion and degradation, may introduce subtle biases into inferences about certain assembly processes [75]. Future research integrating multi-season sampling with long-term hydrological monitoring will be essential to validate the temporal stability of the hierarchical framework reported here and to establish empirically derived water level thresholds capable of informing adaptive management strategies. Within these constraints, the present study provides a conceptual and methodological foundation for understanding how hydrological fluctuations shape community assembly in floodplain lake ecosystems.

## 5. Conclusions

By integrating eDNA metabarcoding with hydroacoustic surveys, this study provides a comprehensive assessment of how winter habitat fragmentation interacts with bathymetric heterogeneity shape aquatic vertebrate community assembly in a large floodplain lake. Our findings reveal a hierarchical assembly framework in which stochastic processes, primarily ecological drift driven by hydrological variability, dominate at the regional scale to shape the species pool, while deterministic filters, particularly water depth, structure local assemblages through trait-based environmental selection. The significant decline in functional richness along the depth gradient, together with vertical size stratification documented by hydroacoustic profiling, confirms that water depth acts as a key local determinant of community structure and niche space. These results collectively implicated that preserving both natural hydrological regimes and local habitat heterogeneity is essential for sustaining the ecological processes that underpin community stability and resilience. As floodplain lakes worldwide face intensifying pressure from anthropogenic hydrological alteration, the hierarchical framework and empirical evidence presented here offer a science-based foundation for environmental flow assessments, habitat conservation, and adaptive management strategies.

## Data Availability

The data that support the findings of this study are available in figshare at https://figshare.com/s/f45476bdbbd6990362ca accessed on 5 March 2026.
